# The effect of adding berry fruit juice concentrates and by-product extract to sugar solution on osmotic dehydration and sensory properties of apples

**DOI:** 10.1007/s13197-019-03658-0

**Published:** 2019-03-04

**Authors:** Kinga Samborska, Lovisa Eliasson, Agata Marzec, Jolanta Kowalska, Dariusz Piotrowski, Andrzej Lenart, Hanna Kowalska

**Affiliations:** 1 Faculty of Food Sciences, Department of Food Engineering and Process Management, Warsaw University of Life Sciences (WULS - SGGW), 159c Nowoursynowska St., 02-776 Warsaw, Poland; 2 RISE Research Institute of Sweden, Agrifood and Bioscience, Box 5401, 402 29 Gothenburg, Sweden; 3 Faculty of Food Sciences, Department of Biotechnology, Microbiology and Food Evaluation, Division of Food Quality Evaluation, Warsaw University of Life Sciences (WULS - SGGW), 159c Nowoursynowska St., 02-776 Warsaw, Poland

**Keywords:** Apple, Bilberry juice, Chokeberry juice, Ethanol bilberry extract, Peleg’s model, By-product

## Abstract

Osmotic dehydration (OD) of apples caused a reduction of normalized water content (NWC) and an increase of normalized solids gain (NSG), independently of the kind of osmotic solution. The use of 22°Brix osmotic solutions with the addition of fruit concentrates or bilberry extract resulted in only slight reduction in the NWC in the samples, i.e. by about 15 and 20%, respectively after 6 and 24 h, against a value up to 80% in case of 65°Brix use. Similarly, larger NSG was achieved at higher solution concentrations, but the differences were smaller. In the case of the use of 80% bilberry press cake extract the NSG was very low but NWC was relatively high. Such a low concentration of slightly concentrated fruit juices is not effective for dehydration of apples, but it may be sufficient to enrich the fruit with the desired colorants. This higher concentration of osmotic solution and a larger addition, especially of the concentrate of chokeberry juice, significantly affected the color changes of dehydrated apples. The apple dehydrated in mixture of 65°Brix sucrose and 15% chokeberry juice concentrate solution exhibited the highest sensory parameters. The addition of berry fruit juices and extract was able to improve the apple sensory quality after 24 h OD in comparison with sucrose solution. Ethanol extract was a good osmotic agent, but not accepted due to taste and overall quality. However, the addition of the extract can be successfully used in conjunction with a sugar solution.

## Introduction

Apples are in great demand on the world market. The main direct of its applying is both fresh and processed apple products. Fruit quality for the market is focused on sensory properties but there is an increasing interest in the health benefits or premium products (alternative to traditional snacks). Consumers are looking for food that tastes, looks attractive and provides nutritional benefits (Spence et al. 2016; Shin et al. 2009). Thus, food industry is very interested in finding the methods to designing new innovative products. In this sense, osmotic dehydration (OD) is a good alternative to create new innovative products with distinctive final properties.

During OD through the semipermeable membrane, two different flows of mass occur; water with solutes (sugars, minerals, pigments, acids and vitamins) from the material to the osmotic solution and, in the opposite direction, from the solution to the food the flow of solute (mainly sugar as osmotic agents) (Park et al. 2002). This mass transfer is important to the composition of the final product (Jiménez-Hernández et al. 2017; Phisut 2012; Tortoe 2010) including its shelf-life and concentration of vitamins (Dermesonlouoglou et al. 2016). Other benefits of osmotic dehydration include effective inhibition of polyphenol oxidase (PPO), prevention of loss of volatile compounds and minimized heat damage to color and sensory properties (Krokida et al. 2001; Saurel et al. 1994).

In recent years, juice concentrates, a natural source of bio-ingredients, have been used as osmotic substances. In addition to reducing the water content in the dehydrated material, it is possible to enrich it. This is very important in terms of creating products high nutritional value and quality. In economic terms, this is particularly important when overproducing fruit. It is preferred to replace sucrose or glucose-fructose syrups, commonly used in dehydration. Taking into account that a relatively high consumption of those substances might have a negative effect on human health, attempts have been made to search for alternatives that can be used for osmotic dehydration. Native sugars and a multitude of other ingredients offer new possibilities for creating attractive fruit-based products (Kowalska et al. 2017a; Lech et al. 2017).

The use of berry fruit juices or extracts from by-products (berry press cake) is new and particularly useful due to the abundance of natural bio-constituents and the wide potential of their use to shape the quality of food, such as color, taste and aroma (Kowalska et al. 2017a). Until now, few studies on the use of juice for dehydration and simultaneous enrichment of food can be found in the literature. There is no information on similar use of by-product extracts.

The need to reduce energy inputs for food processing and strive to develop sustainable technologies puts osmotic dehydration at a high place. This is due to the lack of phase transformation of water, which can be largely removed at moderate temperatures. The fact that osmotic dehydration can be done at low temperature minimizes the damage of the plant tissue and the uptake of sugar has a protective effect on the product reducing or eliminating the need for powerful antioxidants, such as sulfur dioxide (Ciurzyńska et al. 2016). Ethanol extracts from berries press cake can be added to osmotic solutions to enrich. The extract is obtained within the framework of the “green” technologies, i.e. in the supercritical extraction process using carbon dioxide. The supercritical extraction eliminated organic solvent. Kowalska et al. (2017b) demonstrated supercritical extraction which can be used in the recovery of antioxidant compounds from fruit press cake (by-products). Currently, this technology is quite expensive, but that could change if the potential use of such extracts will be noticed. Another aspect is the problem of managing the ever-growing quantities of waste products, which forces to take on new solutions, also economical.

Fruits and vegetables contain bioactive compounds, e.g. anthocyanins, carotenoids discussed for physicochemical characteristics, nutritive values or functional properties (Santos-Buelga and Scalbert 2000; Singh et al. 2016a). Anthocyanins constitute the largest and one of the most important groups of water-soluble natural pigments (Takeoka and Dao 2002). The high value of anthocyanin and other polyphenols were found in many berries such as chokeberry or bilberry. The dark berries such as chokeberry, blueberry and bilberry are well known to be rich in anthocyanins so the choice of juices/extract can be motivated. Infusion of bioactive compound through osmotic treatment into solid food matrix without altering its natural structure has been demonstrated in model food system with watermelon rind with anthocyanin (Bellary et al. 2016). The addition of these compounds into a food tissue could protect them against deteriorating reactions (Betoret et al. 2011). Despite their health-promoting properties, chokeberries are rarely consumed fresh due to their sour, tart and astringent taste. For this reason, the fruit is usually processed and added as an ingredient to other products, e.g. into sweetened beverages. In conclusion the above aspects and the fact that chokeberry and bilberry are well known for their nutritional and beneficial health effects, the choice of juices/extract that can potentially be used during osmotic dehydration is motivated.

The main task of this work was to study the effect of adding berry fruit juice concentrates and by-product extract to sugar solution on osmotic dehydration and sensory properties of apples.

## Materials and methods

### Sample preparation

#### Material

Apples (cv. *Gala*) were obtained from Experimental Field at Warsaw University of Life Sciences, Poland. Fruit was stored in the cold storage room at 4 °C and relative humidity of 85–90% prior to the experiments, however, not longer than 4 weeks. The apples were cut into cylinders having dimension of 10 mm in height and 15 mm in diameters. Then, the apples were submerged in a solution including 0.5% citric acid for 5 min to prevent the enzymatic browning. Apples were blotted on the tissue paper and subjected to osmotic dehydration.

#### Osmotic solutions preparation

The basic hypertonic sucrose solutions were prepared by dissolving commercial sucrose in distilled water at a concentration of 65% (w/w). The addition of chokeberry juice (65°Brix chokeberry *Aronia melanocarpa* juice concentrate), bilberry juice (10°Brix bilberry *Vaccinium myrtillus L.* juice) or extract (80% ethanol bilberry extract from bilberry *Vaccinium myrtillus* press cake) to the sucrose was 5 or 15% (w/w) total mass of solution. Osmotic dehydration of fruit was also carried out in 22% fruit juice solutions.

These osmotic solutions have a high potential for coloring and shaping sensory properties, since they contain, for example, a significant amount of polyphenols, expressed in mg GAE/100 d.m. (GAE means gallic acid), the content of anthocyanins (mg 3-O-β-D cyanidin-glucoside/100 g), the content of flavonoids [mg quercetin/g d.m.] as follows:

1. 80% ethanol bilberry extract coded by “Ex”: 4195/168/51,
2. of 65°Brix sucrose solution and 5% bilberry extract mixture, coded by “S65-5Ex”: 2937/68/46,
3. 65°Brix sucrose solution and 15% bilberry extract mixture, coded by “S65-15Ex”: 7551/270/119,
4. 22°Brix bilberry juice concentrate, coded by “B22”: 4939/1785/48,
5. 65°Brix sucrose solution and 5% bilberry juice concentrate mixture, coded by “S65-5B”: 198/98/2,
6. 65°Brix sucrose solution and 15% bilberry juice concentrate mixture, coded by “S65-B15”: 194/208/3,
7. 65°Brix chokeberry juice concentrate, coded by “Ch65”: 9871/8553/47,
8. 65°Brix sucrose solution and 5% chokeberry juice concentrate mixture, coded by “S65-Ch5”: 790/770/5,
9. 65°Brix sucrose solution and 15% chokeberry juice concentrate mixture, coded by “S65-Ch15”: 2122/2949/8

#### Bilberry extract preparation

Bilberry (*Vaccinium myrtillus*) press cake, a left over from bilberry juice manufacturing, was supplied by Svantes Vilt & Bär (Harads, Sweden). The press cake was produced by cold pressing and had a moisture content of 71% ± 1.2% (w/w) at delivery. The press cake was stored at − 40 °C until use. Extractions were performed in a lab scale system (SFE-500M1-2-C50, Waters, Pittsburgh, USA) equipped in Research Institute of Sweden (Agrifood and Bioscience, Gothenburg) with a carbon dioxide and a co-solvent pump. Just before extraction, 15 g of frozen press cake was milled for 45 s in a mini chopper (C3, Empire Sweden AB, Bromma, Sweden), and then 12 g of the milled press cake was filled in a 100 ml extraction basket of stainless steel. Remaining space of the basket was filled up with glass wool. Extractions were conducted for 60 min at 400 bars and 40 °C. A carbon dioxide flow of 7 g/min and a co-solvent flow of 3 g/min were used. The carbon dioxide had a purity of 4.0 (AGA, Lidingö, Sweden) and the co-solvent was a mixture of ethanol and water (80:20, v/v). Separation of the extract and the carbon dioxide was done in a 500 ml cyclone at 10 bar and 25 °C, and the final extract was collected in falcon tubes. The extraction was repeated five times and then the extract from all replicates were mixed into one single extract that was used for the subsequent osmotic dehydration.

### Osmotic dehydration (OD)

Osmotic treatment of apples was performed at temperature of 45 °C. The basic hypertonic sucrose solutions were prepared by dissolving commercial sucrose in distilled water at a concentration of 65% (w/w). The addition of chokeberry juice (65°Brix chokeberry *Aronia melanocarpa* juice concentrate), bilberry juice (10°Brix bilberry *Vaccinium myrtillus L.* juice) or extract (80% ethanol bilberry extract from bilberry *Vaccinium myrtillus* press cake) to the sucrose was 5 or 15% (w/w) total mass of solution. Osmotic dehydration of fruit was also carried out in 22% fruit juice solutions. The osmotic solutions were used with a solution/fruit mass ratio of 4/1. Samples were collected 0, 0.5, 1.0, 2.0, 4.0, 6.0 and 24 h after the immersion. The process was carried out in a water bath (Water Bath Shaker, Type 357 ELPAN, Poland) with continuous shaking (60 cycles/min). After removal from the solution, the dehydrated apple samples were rinsed in 500 ml of distilled water three times for 3 s and blotted with absorbent paper to remove excess of water. The experiments were conducted in duplicate. Before and after OD process the mass of samples and dry matter content were measured. The dry matter content was determined by the vacuum oven method for 24 h at a temperature of 70 °C and pressure 4 kPa.

### Mass transfer indicators

The following kinetic parameters normalized water content (NWC) and normalized solids content (NSG) as mass transfer indicators were determined using the weight of apple cylinders before and after the osmotic dehydration, as well as dry matter content of apples, before and after OD and calculated according to Eqs. (1) and (2), respectively (Kowalska et al. 2008):

1 $$ NWC = \frac{{\left( {1 - s_{\tau}} \right)\cdot m_{\tau}}}{{\left( {1 - s_{o} } \right)\cdot m_{o} }} $$

2 $$ NSG = \frac{s_{\tau} \cdot m_{\tau}}{{s_{o}\cdot m_{o} }} $$

where m_o_ is the initial fruit mass (g) before OD; m_τ_ is the final fruit mass (g) after time τ of OD; s_o_ is the initial fruit dry solid matter content (g dry matter/g total fruit mass) before OD; s_τ_ is the final fruit dry matter content (g dry matter/g total fruit mass) after time *τ* of pre-treatment. “Normalized water content” means what part of the water remained in the osmotically drained material in relation to its initial content in the raw material, and similarly “normalized solids content” means the share of dry matter in relation to the initial value.

The model proposed by Peleg (1988) was employed to fit the experimental results. It is an empirical model with two parameters (k_1_ and k_2_) initially established to describe curves that approach equilibrium asymptotically. NWC (Eq. 1) and NSG (Eq. 2) data were fitted using Peleg’s model (Kowalska et al. 2008):

3 $$ Y = Y_{o} \mp \frac{\tau }{{k_{1} + k_{2}\cdot \tau }}$$

where Y—means NWC and NSG at time τ, Y_o_ initial value of NWC and NSG (= 1), “+” for NSG and “−" for NWC.

Peleg’s model (Eqs. 1, 2) for both indicators (normalised water content NWC and normalised solids gain NSG) was adequate; in most cases the correlation coefficient was higher than 0.9.

### Color

The color changes of fresh and osmo-dehydrated samples were investigated using Konica-Minolta CR-5 (Osaka, Japan) Chroma Meter according to CIE L*a*b* system. Before analysis the Chroma Meter was calibrated with white and black ceramic standards plates. The measurements were made using CIE L*a*b* scale. L* parameter is lightness, a^*^ parameter indicates red (+) or green (−) parameters of color measurement and b* parameter represents chromaticity yellow (+) or blue (−). On the basis of these parameters total color difference (ΔE), chroma (C) and hue angle (h*) were calculated according to Eqs. (3), (4) and (5), respectively:

4 $$ \Delta E = \sqrt {(\varDelta L^{*} )^{2} + (\varDelta a^{*} )^{2} + (\varDelta b^{*} )^{2} } $$

where ΔL^*^, Δa^*^, Δb^*^ – the change of L^*^, a^*^ and b^*^ parameters between fresh and osmo-dehydrated samples

5 $$ C = \sqrt {\left( {a^{*} } \right)^{2} + \left( {b^{*} } \right)^{2} } $$

6 $$ h = arctan\frac{{b^{*} }}{{a^{*} }} . $$

The analyses were conducted in ten repetitions for randomly selected apple cylinders for each osmotic dehydration condition.

## Sensory evaluation

The sensory evaluation was made by 50 trained consumers (students: 30 females and 20 males, all between 20 and 24 years of age) in laboratory conditions. The following most important characteristics determining the quality and attractiveness of osmo-dehydrated apples were selected: color, taste, softness, flavor and overall quality of fruit were conducted according to the standard ISO PN-EN ISO 13299:2016 (1998) using the method of random sampling. There were the following categories of sensory analysis on a hedonic scale from 0 to 10: color (not regular dark, brown—0, white and creamy like a typical apple or red—10); apple taste (imperceptible—0, very intensive—10); softness (gentle—0, very soft—10); general evaluation of dehydrated food quality (bad—0, very good—10). Samples were coded by 2-digit random numbers in a random order for each panelist. Apples 2 and 24 h osmo-dehydrated in each osmotic medium were subjected to sensory evaluation.

### Statistical analysis

Analysis of variance was conducted using Statistica 12PL and the significant differences between medium values were determined using Duncan’s Multiple Range test at significance level of *p* ≤ 0.05. In the case of an abnormal distribution, division into homogeneous groups was performed using the non-parametric multiple comparison test (Kruskal–Wallis test). An analysis of the Principal Component Analysis (PCA) was also applied in this work (Stanisz 2011).

## Result and discussion

### Osmotic mass transfer indicators

**Fig. 1:**
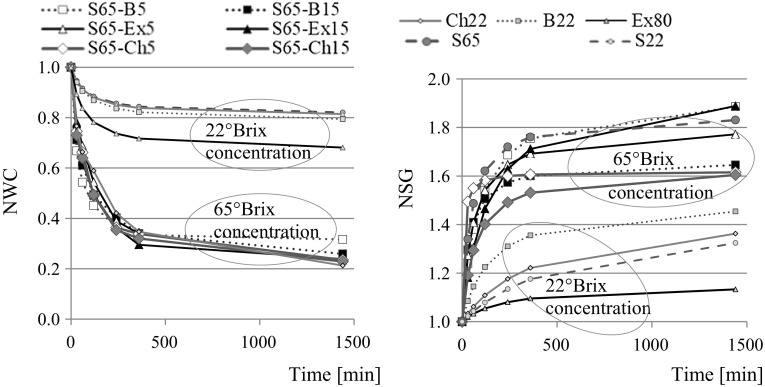
The effect of OD on normalised water content NWC and solids gain NSG in osmo-dehydrated apples by various solutions as in Table 1 and by fitting of Peleg’s model

Osmotic dehydration of apples in sucrose solution and mixture of sucrose and fruit juice concentrate or extract solution caused reduction of normalized water content and increase of normalized solids gain in samples, independently on the kind of osmotic solution (Fig. 1). In the osmotically dehydrated apples in 65°Brix sucrose solutions with the addition of fruit concentrates the normalised water content (NWC) reduction increased during the osmotic process, but mainly to 360 min. After this time the NWC indicator in osmo-dehydrated apples changed very little, especially in samples treated by mixture of sucrose and bilberry juice (from 0.35 to 0.32) (Fig. 1). The most significant changes of water content took place during the first 2 h of OD. After that time normalized water content in apples was reduced by about 48%; from 1.0 to the range of 0.45–0.59, depending on the type of osmotic solution (Fig. 1).

Similar values were obtained in pumpkin osmo-dehydrated in 61.5°Brix sucrose solution at 30 °C for 120 min (Kowalska et al. 2008). The use of a 22°Brix osmotic solution as control samples resulted in a slight reduction in the normalized water content in the samples, i.e. by about 15 and 20%, respectively after 360 and 1440 min. Lower NWC values of around 28 and 35% were noted in apples dehydrated in the ethanol extract of bilberry press cake. Probably such a low concentration of only slightly concentrated fruit juices is not effective for dehydration of apples, but it may be sufficient to enrich the fruit with the desired colorants. Already in 22°Brix berry juice there was a significant increase, by over 40% after 360 min, of the normalized solid gain in the osmo-dehydrated apples (Fig. 1). Lower NSG values were found in apples dehydrated in mixture of sucrose and chokeberry juice concentrate, and the significantly smallest with the bilberry extract. High concentration of osmotic solutions affected several times greater NSG. The values of this indicator in comparison with NWC values were significantly different. A smaller addition of fruit juice to the base sucrose solution resulted in higher NSG values, with the exception of bilberry extract. Significantly lower values were observed in samples dehydrated in a mixture of sucrose and concentrate of chokeberry juice.

### Color parameters

**Fig. 2:**
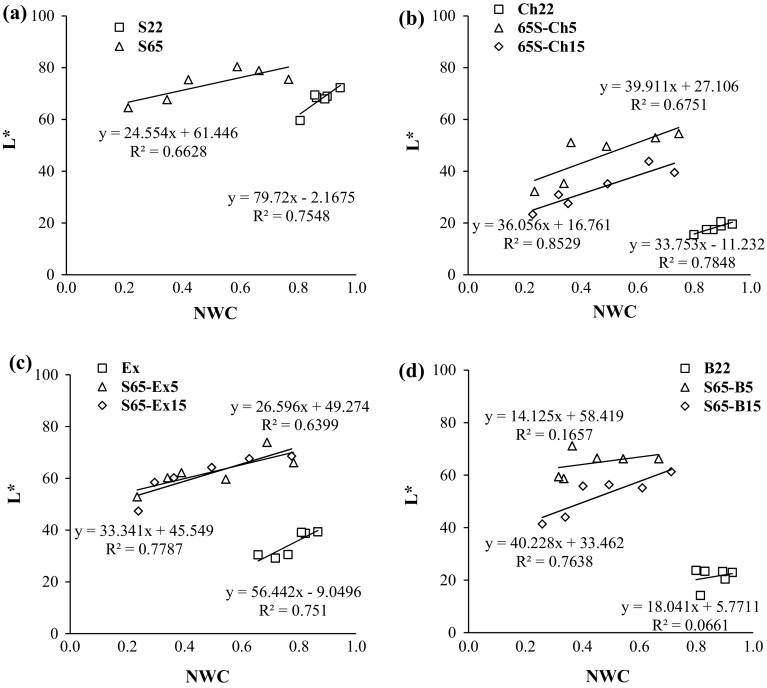
Changes in color lightness (L*) depending on normalized water content NWC for osmo-dehydrated apples by various solutions (coded as in Table 1): **a** 22°Brix (S22) and 65°Brix (S65) sucrose solution; **b** 22°Brix chokeberry juice (Ch22) and 65°Brix mixture of sucrose and 5 (S65–Ch5) or 15% (S65–Ch15) addition of the juice concentrate; **c** 80% ethanol extract from bilberry press cake (Ex) and 65°Brix sucrose solution and 5 (S65–Ex) or 15% (S65–Ex15) addition of the extract; **d** 22°Brix bilberry juice (B22) and 65°Brix mixture of sucrose solution and 5 (S65–B5) or 15% (S65–B15) addition of the juice. Average values were used, standard deviation ranged up to around 5%

The values of the chromatic coordinates for the control samples (fresh apples) (L*, a* and b*) were 77.77 ± 2.09, − 2.69 ± 1.17 and 30.96 ± 4.16, respectively. Compared to fresh fruit osmotic dehydration significantly changed the color of the osmo-dehydrated apples. The type of solution and it concentration, addition of bilberry fruit juices or bilberry extract to sucrose solution had a significant effect on all of the three coordinates (L*, a* and b*) of the osmo-dehydrated apples (Table 1). Addition of berry (chokeberry or bilberry) juices or bilberry extract to sucrose solution during OD caused darkening of fruit samples probably due to the uptake of anthocyanins. With the longer the time and lower the normalized water content there was a decrease in the lightness (L*) of the samples (Fig. 2). Changes of lightness in color of samples positively correlated with normalized water content. In most cases, the correlation coefficient was in the range of 0.64-0.85. Only when using blueberry juice at 22°Brix concentration, there was no correlation (R^2^ = 0.17). Both kind and solute concentration are linked to the color changes, which are verified in dehydrated products. The color alteration in dehydrated apples was less pronounced when the osmotic dehydration occurred at sucrose solution, especially without fruit concentrates or extract addition or when it was lower (5%). The use of color solutions (chokeberry and bilberry concentrates) at 22°Brix resulted in the largest darkening of samples, despite high normalized water content (0.80–0.93). Smaller changes took place in osmotically dehydrated apples in 80% ethanol extract of bilberry fruit press cake. It could be a result in higher uptake of anthocyanin from solution to apple tissue when 22°Brix juice solutions were used (Fig. 2). These changes indicate that osmotic dehydration at 45 °C has not been followed by pigment degradation, which results in higher nutritional quality products. However, there was not a significant effect of type of osmotic solution, its concentration and berry juices or ethanol extract addition on L* value of osmo-dehydrated apples (Table 1).

**Table 1:** Effect of the solution type, concentration and the addition of berry juices or extract to sucrose solution on the color parameters of osmodehydrated apples

	Mass transfer				Color parameters											
	*NWC*		*NSG*		L*		a*		b*		*C*		*ΔE*		*h*	
*Solution type*																
S—sucrose solution, C—chokeberry juice, B—bilberry juice, Ex—bilberry extract, S65-C, S65-B, S65-Ex—mixture of sucrose solution and chokeberry juice, bilberry juice, bilberry extract, respectively																
S22	*p* = 0.000	b	*p* = 0.000	ab	*p* = 0.000	a	*p* = 0.000	b	*p* = 0.000	b	*p* = 0.000	a	*p* = 0.000	c	*p* = 0.000	b
C22		b		ab		b		c		a		c		b		b
B22		b		b		c		a		a		b		b		c
Ex		b		a		d		a		c		b		a		c
S65-C		a		d		e		d		d		d		a		a
S65-B		a		cd		f		a		e		a		d		a
S65-Ex		a		cd		g		e		f		a		e		a
*Solution concentration*																
22°Brix	*p* = 0.000	a	*p* = 0.000	ab	*p* = 0.000	a	*p* = 0.000	b	*p* = 0.000	a	*p* = 0.000	a	*p* = 0.000	a	*p* = 0.000	a
65°Brix		b		d		b		a		b		b		b		b
Ex		a		a		a		a		c		a		c		a
*Addition of berry juices or extract to sucrose solution*																
0%	*p* = 0.000	a	*p* = 0.000	d	*p* = 0.000	a	*p* = 0.000	a	*p* = 0.000	a	*p* = 0.152	a	*p* = 0.000	a	*p* = 0.000	a
5%		c		cd		b		b		b		b		b		b
15%		b		cd		c		c		c		b		c		b

Average values were used, standard deviation ranged up to around 5%

a, b, c—homogenous group

*p* < 0.05

**Fig. 3:**
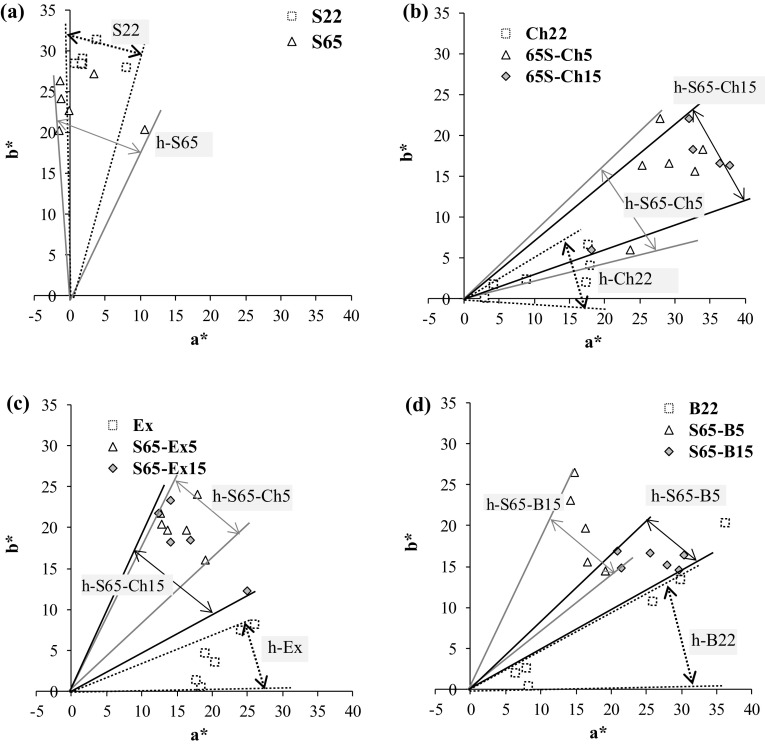
Changes in a*, b* and h indicators of osmo-dehydrated apples (coded as in Table 1): **a** 22°Brix (S22) and 65°Brix (S65) sucrose solution; **b** 22°Brix chokeberry juice (Ch22) and 65°Brix mixture of sucrose solution and 5 (S65–Ch5) or 15% (S65–Ch15) addition of the juice concentrate; **c** 80% ethanol extract from bilberry press cake (Ex) and 65°Brix mixture of sucrose solution and 5 (S65–Ex5) or 15% (S65–Ex15) addition of the extract; **d** 22°Brix bilberry juice (B22) and 65°Brix mixture of sucrose solution and 5 (S65–B5) or 15% (S65–B15) addition of the juice. Average values were used, standard deviation ranged up to around 5%

The h parameter (hue angle), which determines the color position on the color wheel, showed various values, depending on the type of solution that was used (Fig. 3). It can also be correlated to a higher transfer of anthocyanins from fruits into the osmotic solutions (Montes et al. 2005).

The hue (shade) of the color measured in the samples dehydrated in the range 0–1440 min changed from the green-yellow color [samples dehydrated in sucrose solutions (Fig. 3a)] towards the red shade in a gentle manner [samples dehydrated in the extract solution or to a lesser degree, in the sucrose solution with the addition of extract (Fig. 3c)], through the more red [samples dehydrated in bilberry juice or to a lesser degree in the sucrose solution with the addition of the juice (Fig. 3d)] to the more red one, in the dehydrated in the concentrate chokeberry or juice solutions (Fig. 3c).

**Fig. 4:**
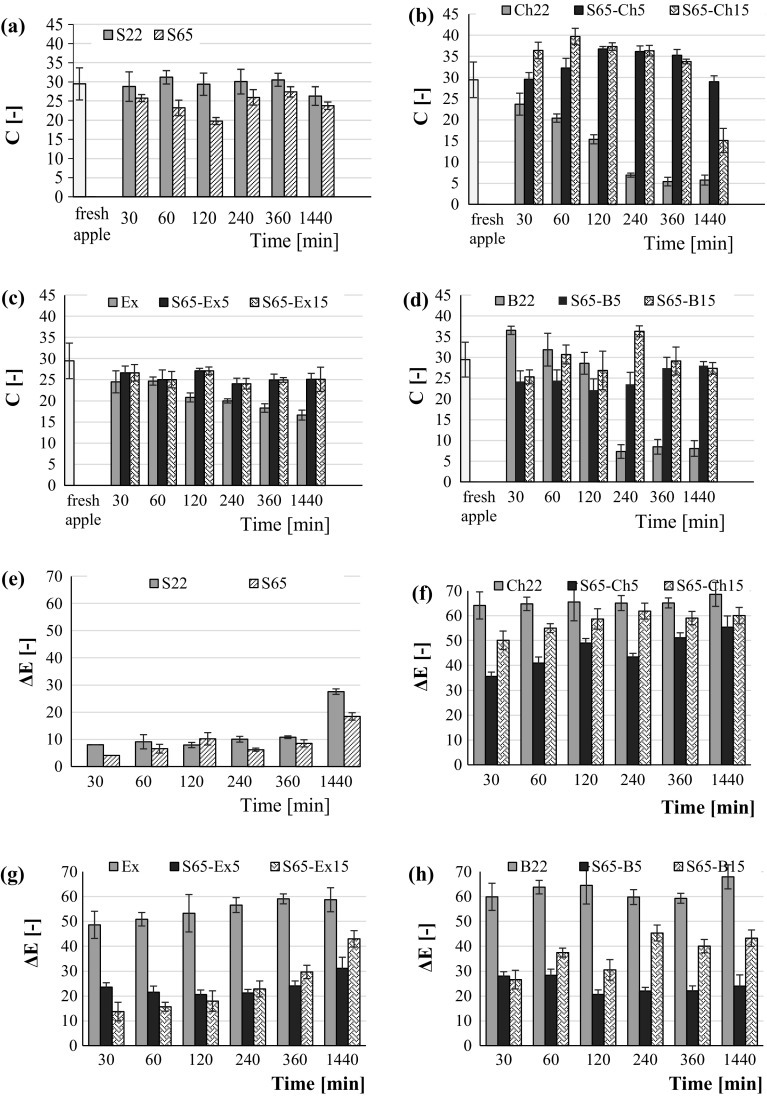
Chroma (C) and total color change (ΔE) of osmo-dehydrated apples (coded as in Table 1): **a**, **e** 22°Brix (S22) and 65°Brix (S65) sucrose solution; **b**, **f** 22°Brix chokeberry juice (Ch22) and 65°Brix mixture of sucrose solution and 5 (S65–Ch5) or 15% (S65–Ch15) addition of the juice concentrate; **c**, **g** 80% ethanol extract from bilberry press cake (Ex) and 65°Brix mixture of sucrose solution and 5 (S65–Ex) or 15% (S65–Fx15) addition of the extract; **d**, **h** 22°Brix bilberry juice (B22) and 65°Brix mixture of sucrose solution and 5 (S65–B5) or 15% (S65–B15) addition of the juice. The ΔE of samples was calculated with reference to the fresh apple tissue

The type solution, concentration and addition of berry juices or extract to sucrose solution had a significant effect on the chroma parameter C (color saturation) (Fig. 4a–d, Table 1). However there was no difference in color saturation of apple dehydrated in 22°Brix sucrose, mixture of 65°Brix sucrose and bilberry juice or extract (homogenous group) (Table 1). In apple dehydrated in 22°Brix chokeberry juice the highest decrease of chroma C was observed. It could be related to enzymatic and non-enzymatic browning while impregnation of pigment substances from solution to apple tissue during OD. Non-enzymatic browning may be a result from condensation of carbonyl group with amino acids, reactions of sugars and ascorbic acid in the absence of free amino acids (Valdramidis et al. 2010). Probably irregular changes in color parameters of dehydrated apple were caused both by uptake of anthocyanins and enzymatic/nonenzymatic browning at the same time. In 65°Brix sugar solution with addition of bilberry extract, irregular changes in color saturation C was not observed. Probably it was due to denaturation of proteins (enzymes) by ethanol bilberry extract, and thus an inhibition of enzymatic browning.

High temperature increases rate of mass transfer during OD (Kowalska et al. 2017a) and also enzymatic changes as well as chemical ones. Optimum temperature activity of many enzymes is in the range 20–30 °C. The use of the low temperatures associated with the protector effect that sugar has in some pigments can minimize these alterations during OD process (Landim et al. 2016).The fruit pieces surrounded by sucrose solution are prevented against enzymatic and oxidative browning. Garcia-Noguera et al. (2014) showed that osmotic dehydration enhanced the color in strawberry samples, especially after prolonged ultrasonic exposure and using high sucrose concentrations. Previously subjected to ultrasound and OD freeze-dried strawberry presented a more vivid color and red than the fresh and non-treated strawberries.

Sugars and sugar degradation products are capable of reacting with the anthocyanins of fruit to increase their rate of loss (Francis 1989). Several substances such as furfural and hydroxymethylfurfural formed during the browning reaction also may cause color loss in strawberry products. These compounds are able to reacting with pelargonidin 3-glucoside forming yellow chalcone species. There would be a result in an increase in the hue value (Yang et al. 2008). Enzymatic browning occurs when enzymes, present naturally in fruit such as peroxidase and polyphenol oxidase, catalyze the oxidation of phenols to orthoquinones in the presence of oxygen. Orthoquinones produce brown pigments (melanins). Ascorbic acid causes a reduction in enzymatic browning but does not inhibit enzymatic activity directly. It acts as a reducing compound and reduces the orthoquinones to dehydroxyphenols (Verma et al. 2014).

The total color change (ΔE) of osmo-dehydrated apple was calculated using as reference the fresh sample (Fig. 4e–h). The longer the OD time and the addition of berry fruit juices or extract increase caused higher total color change and allowed to distinguish sample groups with different hue color (Fig. 3). Moreover, the type solution and addition of berry juices or extract to sucrose solution was found as statistically significant (Table 1). Apple samples dehydrated in sucrose solution characterized the smallest value of total color change, especially to 360 min of OD (Fig. 4e–h). Furthermore, the higher ΔE with using lower concentrated sucrose solution was observed. The total color change strongly increased with the addition of berry fruit juices or extract to sucrose solution. The use of the color measurement provided a means of quantifying color changes in osmo-dehydrated apple and can help in designing a final color of product.

### Sensory evaluation

**Fig. 5:**
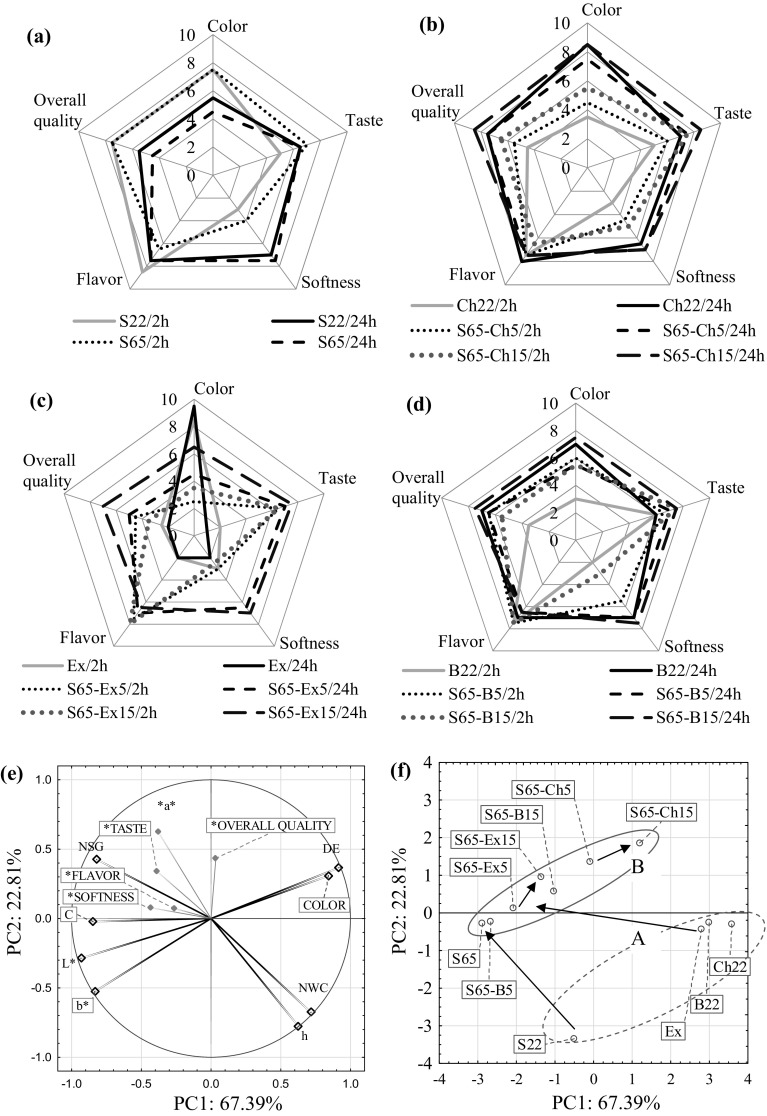
Sensory properties of osmo-dehydrated apples (coded as in Table 1): **a** 22°Brix (S22) and 65°Brix (S65) sucrose solution; **b** 22°Brix chokeberry juice (Ch22) and 65°Brix mixture of sucrose solution and 5 (S65–Ch5) or 15% (S65–Ch15) addition of the juice concentrate; **c** 80% ethanol extract from bilberry press cake (Ex) and 65°Brix mixture of sucrose solution and 5 (S65–Ex) or 15% (S65–Fx15) addition of the extract; **d** 22°Brix bilberry juice (B22) and 65°Brix mixture of sucrose solution and 5 (S65–B5) or 15% (S65–B15) addition of bilberry juice. Diagram PCA: **e** in a plane factors, **f** for osmo-dehydrated apple for 24 h by various solutions (as in Table 1); the arrows indicate the directions of changes in PC 1 or PC 2 values depend on solution concentrates or higher proportion of juices or extract addition. Average values were used, standard deviation ranged up to around 5%

Sensory results were grouped around complex sensory properties such as color, taste, flavor, softness and overall quality (Fig. 5a–d). As regards the appearance color scores ranging from 9.5 (apple dehydrated in 80% ethanol extract from bilberry press cake after 24 h OD) to 3.0 (apple dehydrated in 22°Brix bilberry juice after 24 h OD) were obtained. Significant differences (p < 0.05) were found for this discriminant of sensory quality. This was confirmed by the above-discussed results of the instrumental measurements of the color parameters.

The osmo-dehydrated samples after 24 h of OD had a better overall quality than the samples after 2 h of OD (Fig. 5a–d). Apples dehydrated in 80% ethanol extract of bilberry press cake were imperceptible and not soft in comparison with the rest of osmo-dehydrated apple samples. Imperceptible taste of apple was probably caused by high solubility of carbohydrates in ethanol, and thus removal of carbohydrates from apple tissue. The literature evidenced that ketos sugars (fructose) show higher solubilities in ethanol than aldoses (glucose). Additionally, the solubility of common sugars in ethanol–water mixtures is also available in the research works (Montañés et al. 2007). However, the addition of a bilberry extract to sucrose solution has improved the taste and softness of osmo-dehydrated apples for 2 and 24 h. Finally, in terms of overall quality, the highest score was achieved by addition of 5 and 15% chokeberry juice to sucrose solution. Products were characterized by quite good color (7.5 and 8.0, respectively) and good taste (7.5 and 8.0, respectively). In addition, the high overall quality of osmo-dehydrated apples in a mixture of sucrose solution and chokeberry juice could affect the aromatic character of chokeberry. Chokeberry fruit is characterized by a tart and bitter taste caused by a high content of polyphenols. Therefore, they are rarely used for direct consumption (Santos-Buelga and Scalbert 2000). A product that is important and valuable for industry is chokeberry fruit juice, which is an excellent material for processing, with high bioactive potential, which could be used as a flavoring additive and a pigment, enriching the quality of the final products. The proportion of sour and sweet taste played a significant role in the sensory evaluation of food (Teleszko and Wojdyło 2014). Consuming food containing bio-components, mainly polyphenols, is beneficial due to other aspects. Singh et al. (2016b), on the example of jambolan fruit, observed significant variability of antioxidant and antimicrobial activity.

A principal component analysis (PCA) with classification was conducted taking into account mean values of individual parameters (Fig. 5e). Types of osmotic solution in the aspect of mass transfer (NWC, NSG), the color parameters and sensory properties of apples were taken into account. All analyzed parameters of osmo-dehydrated apples after 24 h took part in the analysis. By using the sufficient proportion criterion and Kaiser’s criterion (Stanisz 2011), the number of the analyzed variables was reduced to two principal components (PC 1 and PC 2). The PC 1 component included the instrumental color parameters (L*, b*, ΔE, C, h) and color as sensory descriptor (Fig. 5e–f). The PC 2 component included the instrumental color parameter a* and sensory descriptors (taste, softness, flavor and overall quality) (Fig. 5e). The selected components PC 1 and PC 2 explained 91.63% of the variability of the analyzed properties of osmo-dehydrated apples (Fig. 5e) with simultaneous 8.37% loss of information, respectively. A significant effect of changes in the normalized water content of NWC during the OD on color parameters was found (correlation higher than 0.5). Its value obtained in samples dehydrated in different osmotic solutions for 24 h positively correlated with the hue h and, with minus correlation, color saturation C, parameter a* and L*. More important was normalized solids gain NSG, which positively correlated with brightness L*, chroma C, taste and softness but negatively with total color change ΔE, color hue h, color as sensory descriptor. Changes related to mass transfer in dehydrated samples, i.e. reduction of water content NWC and penetration of osmotic substances NSG, have a direct effect on color and sensory descriptions. The application of a low concentration of osmotic solution, e.g. sucrose, may be associated with too little protection against darkening of the samples. However, a higher proportion of juices, its concentrates or extract in the osmotic solution affects the larger color changes. In the figure, the directions of changes in PCA 1 and PCA 2, depending on the increase in the concentration of the osmotic solution and the share of additives, have been appropriately marked with arrows. The analysis of the obtained results showed that overall quality positively correlated only with taste and softness. Moreover, softness, flavor and overall quality high correlated with taste. Instrumentally appointed color parameters were very important and higher total color change (ΔE) associated with a higher sensory evaluation with regard to color. However, the changes involving the darkening of the color were perceived negatively. Singh et al. (2016a, b) evaluated the correlation of Indian fruits and vegetables properties with their color parameters by PCA method. According to the study L*, a* and b* values of different plant tissue were related positively with ash and fat content, but negatively with protein content.

Principal component analysis (PCA) revealed two groups (A, B) of apples with different color and sensory properties (Fig. 5f). Basically, two concentration values of osmotic solutions, 22 and 65°Brix, were applied to the distribution of samples. As part of the dehydrated group A in a lower concentration of solutions, the samples dehydrated in the sucrose solution, which color brightness L* has changed to the smallest degree, are significantly different from group B, i.e. samples dehydrated in a higher concentration, which color brightness L* has changed to the most.

The analysis of the obtained results showed that both color and sensory properties have a significant effect upon the perception of osmo-dehydrated apples by consumers. The correlations found indicate that type of osmotic solution with fruit juice addition could be used as osmotic substance, mainly to water content reduction—solid gain increase, and also to change color of product as well as good taste and flavor.

## Conclusion

Presented results indicate that it is possible to design the color and other sensory attributes of apples during osmotic dehydration by using osmotic solutions containing different proportion of sugar, chokeberry and bilberry juice concentrates or ethanol bilberry extract. The osmotic dehydration in the sugar solution with addition of berry fruit juice concentrates and by-product extract reduces the water content and increases solids gain, to changing color, taste, softness and flavor of processed apples. The addition of 5 and 15% of berry fruit juices or extract to sucrose solution causes color changes in semi-dehydrated product. Addition of 15% of chokeberry juice to sucrose solution assured the highest sensory parameters, including color, taste and overall quality of processed apples. The worst product was obtained after osmotic dehydration of apples in ethanol extract from bilberry press cake. However, the addition of extract from bilberry press cake can be successfully used in conjunction with a sugar solution.
